# Factors That Predict Food Skills in Canadian Gym Members: A National Cross-Sectional Survey

**DOI:** 10.3390/nu15194118

**Published:** 2023-09-23

**Authors:** Courtney Barlott, Candace Cunningham, Kristina Miller, Paula D. N. Dworatzek

**Affiliations:** 1 School of Food & Nutritional Sciences, Brescia University College, Western University, 1285 Western Rd., London, ON N6G 1H2, Canada; barlottc@gmail.com (C.B.); ccunningham@hpph.ca (C.C.); 2 Formerly of GoodLife Fitness, 710 Proudfoot Ln, London, ON N6H 1T2, Canada; kmille46@gmail.com; 3 Schulich Interfaculty Program in Public Health, Schulich School of Medicine and Dentistry, Western University, 1151 Richmond St., London, ON N6A 3K7, Canada

**Keywords:** food skills, food literacy, cooking skills, gym members, cross-sectional survey, nutrition beliefs, food experiences, food behaviours

## Abstract

This study determined predictors of food skills in Canadian gym members. A random sample of gym members were invited to complete a validated Food Skills Questionnaire with supplementary questions. All questions/variables significantly associated (*p* < 0.05) and fair-to-moderately correlated (r ≥ 0.40) with Total Food Skills (TFSs) were analyzed by multiple regression. The respondents’ (*n* = 576) mean ± SD age was 41.3 ± 14.8 years, with 67.3% females and 13.2% students. The mean TFSs score was 77.1 ± 11.9 (maximum 100). Females reported higher TFSs than males; however, this did not remain significant when nutrition-related beliefs were considered. Increasing age, taking a nutrition/cooking course, teen meal preparation, primary cook, time preparing weekend meals, believing that preparing healthy food is important, and self-reported nutritional quality of diet and nutrition knowledge were positively associated with TFSs (*p* < 0.05). Purchasing food/beverages from convenience stores, buying pre-prepared dinners, and being a student were negatively associated with TFSs (*p* < 0.05). The strongest predictors of TFSs were self-reported nutrition knowledge and nutritional quality of diet. The adjusted R^2^ increased by 0.30 when food-related experiences/behaviours and nutrition-related beliefs were included in the final model, which accounted for 50% of the variance in TFSs. Food experiences/behaviours and nutrition beliefs, which are associated with food skills, are potential intermediary targets for programs and/or research to improve food skills.

## 1. Introduction

How we buy, prepare, and consume food has changed significantly in the last several decades. The increased industrialization of the food system has resulted in an abundant supply of processed and convenient foods, with the result that fat, sodium, and sugar intakes have become excessive in the diets of Canadians [1,2]. Simultaneously, lower intakes of fruits, vegetables, and whole grains have also been reported [3]. Canadians tend to eat away from home more often and eat together less often than in the past [4,5]. Alongside these changes, there have been reported declines in food literacy [6,7,8]. Conversely, involvement in cooking and developing adequate food skills have been associated with healthier eating habits, mostly due to the greater use of vegetables and minimally-processed ingredients [7,8,9,10,11].

Food literacy, food skills, and cooking skills have all been used to describe this area of study. Definitions have emerged but are still varied [12,13,14,15,16]. Food literacy tends to have a broader definition than food skills, which is broader than cooking skills. Food literacy has been suggested to encompass five interconnected categories: food and nutrition knowledge; food skills; self-efficacy and confidence; food decisions; and ecological (external) factors [12]. It has been defined as “an understanding of the full cycle of food practices (farm to table to waste), including impacts on health, and economic, social, and environmental sustainability” [13]. Food skills have a narrower definition, defined as a “complex, interrelated, and person-centered set of skills…needed to provide and prepare safe, nutritious, and culturally acceptable meals for all members of a household” [17].

The recent literature suggests food skills are an important component of food literacy because food skills were found to be associated with diet quality, while cooking skills were not [18]. It is preferable to define food skills more broadly as encompassing cooking skills as well as a variety of other components, such as planning, budgeting, shopping, and food safety [13]. Food literacy and food skills have been difficult to measure, but more recently, progress has been made [13,14,16,19,20]. A previously validated Food Skills Questionnaire (FSQ) allocates skills into the following three domains: Food Selection and Planning, Food Preparation, and Food Safety and Storage [13,21]. Within each domain, knowledge, conceptualizations, attitudes, and behaviours can be captured [13,21]. The FSQ was developed and validated to assess basic to intermediate food skills [13]; however, in a population with a more diverse age range, more complex skills such as creating new recipes from scratch, canning and freezing food, and composting food waste [22], should be considered.

Many previous studies on food skills have focused on university students and young adults [10,13,23,24,25,26], with results suggesting that increasing age [24,25], female gender [10,24,25], independent living situations [25], and having taken a food/nutrition course [23,24,25,26] were associated with higher food skills. This study looks to expand on this work by assessing food skills in a more diverse group of adult Canadians who hold gym memberships. More specifically, this study will assess food skills in adult Canadian gym members and determine if food skills are associated with or predicted by demographics, lifestyle, food-related experiences and behaviours, and nutrition-related beliefs.

## 2. Materials and Methods

This cross-sectional observational study was conducted with Canadian GoodLife Fitness members. Goodlife is a Canadian fitness club with over 1.5 million members [27]. Recruitment through Goodlife Fitness allowed for recruitment of individuals who are generally interested in maintaining their health from a diverse group of Canadians in provinces all across Canada (except Quebec). In February 2020, 30,000 members were randomly selected to receive an email invitation for an online survey assessing food skills and its potential predictors. The sample was chosen from current gym members who were 18 years or older, able to read and write in English, and consented to receive emails from Goodlife. A sample size calculation determined that a minimum sample size of 385 members was needed to have sufficient statistical power to allow for generalizability to Canadians who have gym memberships. In addition, this sample size allows for multiple regression, where the sample size must be at least ten times the number of predictor variables included in the model [28]. Consent was indicated when emailed members entered the survey; however, respondents could skip or refuse to answer any question (as required by ethics), resulting in varying sample sizes for each survey item. The survey closed two weeks after the initial email invitation. A Dillman method of reminders [29] was not used as Goodlife communication protocols do not allow for multiple emails. Members were invited to enter their name into a draw at the end of the survey for one of five CAD 100 cash prizes. The data were anonymized by Goodlife and provided to the researchers. The Non-Medical Research Ethics Board at Western University reviewed and approved this study (#113709).

Using Verint Enterprise (Verint Systems Inc., Melville, NY), the online survey consisted of 83 items, predominantly closed-ended categorical and scaled-response questions. Food skills were assessed using the 39-item previously validated and reliable FSQ [13], with the addition of eight questions (Table 1) to assess more advanced food skills [22]. Three questions were based on number of days per week (0–7), and one question was a 4-point scale (Table 1); these questions were re-weighted to an 11-point scale. For all the remaining questions, respondents rated their food skills on an 11-point scale from 0 to 100, with a higher score indicating a higher proficiency. Such 11-point percent-based scales have been recommended and used effectively in measuring self-reported behaviours and attitudes [13,30]. They also have the advantage that they have equal intervals between successive points (while most Likert-like scales do not). They have been shown to reduce skew and kurtosis and approximate a normal distribution, which allows for analysis as a continuous variable [31,32]. Questions assessing food skills were categorized into three domains: Food Selection and Planning, Food Preparation, and Food Safety and Storage. Food Selection and Planning included skills such as budgeting, meal planning, using a grocery list when shopping, reading food labels, and checking the best before date (Table 1). Food Preparation included skills such as the ability to and frequency of making home-prepared meals and confidence in baking, boiling, steaming, stewing, pan- and stir-frying, and using vegetables or beans and lentils when cooking. Food Safety and Storage captured skills such as cooking food to the correct internal temperature, washing hands and countertops before preparing food, and putting leftovers in the fridge within 2 h. There is currently no gold standard for the assessment of actual food skills, and it has been noted in the literature that self-reported data is appropriate as an individuals’ self-perceived abilities influence their motivation and utilization of food skills [6,13,33,34].

The remaining 36 non-food, skill-related items were as follows: demographics such as age, gender, student (full- or part-time), income, number of children, and education; lifestyle factors such as physical activity, employment, and commute time; food-related experiences such as being a primary cook, frequency of meal preparation as a teenager, having taken a nutrition/cooking course, and having access to a kitchen; food-related behaviours such as purchasing food/beverages at grocery stores, restaurants, and convenience stores, consuming pre-prepared foods, and frequency of grocery shopping; and nutrition-related beliefs such as preparing healthy food is important, and self-reported nutritional quality of diet and nutrition knowledge.

The data were checked for completeness and normality prior to statistical analysis. Respondents were included in the analysis if they answered at least one question and met all inclusion criteria. A food skills score was calculated for each domain if a respondent answered 50% or more items in the domain, and total food skills (TFSs) were calculated as a weighted average of the 3 domains.

The data were analyzed using IBM SPSS Statistics, version 26.0 (IBM Corp., Armonk, NY, USA, 2019). Continuous variables (i.e., food skills, age, importance of preparing healthy food, nutritional quality of diet, and nutrition knowledge) were summarized as either mean and standard deviation (mean ± SD) or as median and interquartile range (mean ± SD presented for consistency), while categorical variables were summarized as percentages. Age was presented as both a continuous variable and a categorical variable representative of young, middle-aged, and older adults. The categorical version was used in the bivariate and regression analyses. Bivariate analyses were conducted to determine associations between independent variables and the primary outcome variable, TFSs. Categorical independent variables were analyzed using Independent Samples *t*-test or One-way ANOVA, and continuous independent variables were analyzed by Pearson’s correlation. (Non-parametric tests (e.g., Spearman’s Rho) were utilized if the data were not normally distributed.) In all analyses a *p*-value of <0.05 was considered statistically significant.

All independent variables that were significantly associated (*p* < 0.05) and fair-to-moderately correlated (r ≥ 0.40) with TFSs were entered into a preliminary stepwise multiple regression model [35]. Twenty-five variables met these criteria and were included in the stepwise regression, and eleven variables remained significant. The cut-point significance level specified for the stepwise regression was P(r) = 0.10. Gender did not remain significant in the stepwise regression model but it was included, together with the 11 variables, in a blockwise regression model as gender has been previously associated with food skills [7,8,18,22,24,25,36] and is an important demographic variable. All regression models were checked for normality, collinearity, and power. The 12 variables were sorted into the following blocks: Model 1 included demographic characteristics (age, gender, and student); Model 2 included variables related to food experiences (teen meal preparation, primary cook, and nutrition/cooking course); Model 3 included variables related to food behaviours (purchasing food/beverages at convenience stores, buying pre-prepared dinner, and spending time preparing meals on weekends); and Model 4 included variables related to nutrition beliefs (preparing healthy food is important, nutritional quality of diet, and nutrition knowledge).

## 3. Results

In total, 583 members completed the survey. Seven respondents who self-identified as under eighteen years were excluded, reducing the sample size to 576. As questions were required to be skippable, sample sizes varied; the lowest sample size for any item was *n* = 529 (92% of respondents). Specific sample sizes are provided in tables. Two questions (access to a kitchen; and sufficient access to kitchen utensils, equipment, and appliances) were not analyzed because all but two respondents answered ‘yes’.

Demographic and lifestyle characteristics are included in Table 2. The mean age of respondents was 41.3 ± 14.8 (mean ± SD) years, and more respondents were female (67.3%). Those that identified as ≥55 years or female had significantly higher food skills in all three domains than those who did not (*p* < 0.001 for all). TFSs were higher in respondents with higher incomes, education levels, those living independently from parents, and those with children. The majority of respondents (81.4%) reported they were a primary cook in their household, with more primary cooks identifying as female (71.8%). Primary cooks and those who had taken a nutrition/cooking course had higher food skills in all domains and TFSs than those who were/have not (*p* < 0.001 for all). Respondents who reported higher frequency of meal preparation as a teenager also had higher TFSs (*p* < 0.001), with higher Food Selection and Planning (*p* = 0.002) and Food Preparation scores (*p* < 0.001), but Food Safety and Storage was not significantly different. Respondents who reported purchasing foods/beverages from convenience stores, and those purchasing more food/beverages from restaurants/fast food/coffee shops had significantly lower food skills across all domains and TFSs (*p* < 0.005 or less for all). Similarly, those who indicated buying pre-prepared breakfast, lunch, or dinner ≥2 times/week had lower TFSs (*p* < 0.001 for all). Conversely, those who reported spending more time preparing meals on weekdays and weekend days had higher TFSs (*p* < 0.001). With respect to physical activity, those who reported exercising ≥150 min/week had higher TFSs (*p* = 0.003), specifically Food Selection and Planning (*p* = 0.001) and Food Preparation (*p* < 0.001) scores, compared with those exercising <90 min/week. Most respondents believed that preparing healthy food is important (88.3 ± 14.2), with females indicating it was significantly more important compared with males (90.2 ± 12.4 vs. 84.0 ± 16.7, respectively, *p* < 0.001).

### 3.1. Domain Food Skills

The item and domain scores can be found in Table 1.

#### 3.1.1. Food Selection and Planning Domain

The mean ± SD for the Food Selection and Planning domain was 76.0 ± 14.5. The lowest score was for creating new recipes from scratch (53.9 ± 33.8), while the highest score was checking best before dates (87.3 ± 17.8). Respondents who reported being a primary cook had significantly higher scores for all skills within this domain compared with those who did not (*p* < 0.05). Respondents who had taken a nutrition/cooking course had significantly higher scores for most skills (*p* < 0.01), except for checking best before dates, using a grocery list, and purchasing a variety of vegetables. Females reported significantly higher scores than males for most skills (*p* < 0.05), except for meal planning, budgeting for groceries, reading food labels, and creating new recipes from scratch, which were not significantly different.

#### 3.1.2. Food Preparation Domain

The mean ± SD for this domain was 78.5 ± 13.5. The lowest score was for ‘food preparation takes too much time’ (reverse coded; 58.5 ± 29.9), and the highest score was ‘for describe your overall ability to prepare meals’ (95.1 ± 13.0). Primary cooks had significantly higher scores for most skills compared with those who were not (*p* < 0.01), except for ‘making meals at home helps me eat healthier’, which was not significantly different between the groups. Respondents who had taken a nutrition/cooking course had significantly higher scores for most skills compared with those who had not (*p* < 0.05), except for ‘making home-prepared meals at breakfast’ and ‘making meals at home helps me eat healthier’, which were not significantly different. Females rated their skills higher in all areas of food preparation (*p* < 0.05), compared with males, except for the following which were not significantly different: baking, grilling, and roasting; choosing a spice or herb; using knives; and peeling, chopping, and slicing.

#### 3.1.3. Food Safety and Storage Domain

The mean ± SD for this domain was 75.8 ± 12.8. Respondents’ confidence in canning ingredients into sealed jars was particularly low (30.8 ± 31.6), while the highest score was for washing hands before preparing food (92.5 ± 14.0). Primary cooks scored significantly higher on most skills (*p* < 0.05), except for canning ingredients, composting fruits/vegetables, dating and labelling stored food, and keeping raw meat separate from foods that will not be cooked. Those who had taken a nutrition/cooking course reported significantly higher scores for most skills than those who had not (*p* < 0.05), except for the following: washing countertops before preparing food; washing fruits/vegetables; composting fruits/vegetables; putting leftovers in the refrigerator within 2 h; and dating and labelling stored food. Females reported significantly higher scores than males for all skills (*p* < 0.05), except for canning ingredients, staying safe in the kitchen, and following the instructions for storage on packaged food.

### 3.2. Total Food Skills

The TFSs analyses can be found in Table 2. The mean ± SD for TFSs was 77.1 ± 11.9, with females having significantly higher TFSs than males (78.9 ± 11.2 vs. 73.4 ± 12.6, respectively; *p* < 0.001). A significantly higher proportion of females reported that they were a primary cook, compared with males (86.9% vs. 69.9%, respectively; *p* < 0.001). Respondents who reported they were a primary cook had significantly higher TFSs than those who were not (79.0 ± 11.1 vs. 68.8 ± 12.0, respectively; *p* < 0.001). Additionally, those who had taken a nutrition/cooking course (39.1%) had significantly higher TFSs than those who had not (80.7 ± 10.9 vs. 74.7 ± 12.0, respectively; *p* < 0.001). Females who had taken a nutrition/cooking course had significantly higher TFSs than females who had not (82.9 ± 10.2 vs. 75.8 ± 11.1 respectively, *p* < 0.001); this difference was not seen in males.

Respondents involved in meal preparation as a teenager four or more times/week (19.0%) had significantly higher TFSs compared with those involved less than once a week (80.8 ± 11.1 vs. 75.2 ± 12.6, respectively; *p* < 0.001). There were no significant differences in the frequency of meal preparation as a teenager by gender (*p* = 0.22).

TFSs scores were significantly higher with increasing age categories, with respondents aged ≥55 years having the highest food skills (82.5 ± 11.3), followed by those 35 to 54 years (78.0 ± 11.6) and 18 to 34 years (73.5 ± 11.4) (*p* < 0.001). Respondents who identified as a student (13.2%) had significantly lower TFSs compared with those who did not (73.5± 13.3 vs. 77.6 ± 11.7, respectively; *p* = 0.005). Females who did not work had significantly higher TFSs compared with females who did (83.9 ± 10.9 vs. 77.7 ± 11.0, respectively; *p* < 0.001); however, this difference was not seen in males. There was no significant difference between TFSs in females with or without children (*p* = 0.18), but males with children had significantly higher TFSs than males without children (77.7 ± 9.2 and 72.0 ± 13.3, respectively; *p* < 0.002). Respondents who exercised ≥150 min/week (55.9%) had significantly higher TFSs, compared with those who exercised <90 min/week (78.4 ± 12.4 vs. 73.8 ± 11.2, respectively; *p* = 0.003).

TFSs were correlated with the self-reported nutritional quality of diets (r = 0.56; *p* < 0.001) and nutrition knowledge (r = 0.58; *p* < 0.001; Table 3). There were no significant differences in the following independent variables when performing bivariate analyses with the TFSs score as the dependent variable: part-time vs. full-time students, province, length of commute, time to nearest grocery store, number of children, amount of paid work/week, number of meals and snacks eaten/day, and frequency of making own meals.

### 3.3. Predictors of Total Food Skills

When all 25 significant variables (Table 2 and Table 3) were included in the stepwise regression, 11 variables remained significant. Gender did not remain significant, but due to its previously identified association with food skills [7,8,18,22,24,25,32] and importance as a demographic variable, it was included in the blockwise regression, for a total of 12 variables (Table 4).

In Model 1, increasing age was positively associated with TFSs (*p* < 0.001), males had significantly lower food skills than females (*p* < 0.001), and being a student was not significantly associated with TFSs. These three variables accounted for 9.0% (Adjusted R^2^ = 0.09) of the variance seen in TFSs.

In Model 2, all three variables related to food experiences (primary cook, teen meal preparation, and nutrition/cooking course) were positively associated with TFSs (*p* < 0.001). The standardized betas for primary cook and age (0.24 and 0.22, respectively) revealed that these two variables had the largest relative influences on TFSs, followed by teen meal preparation (0.17), having taken a nutrition/cooking course (0.16) and being male (−0.11). Model 2 explained an additional 11% (Adjusted R^2^ = 0.20) of the variance seen in TFSs.

When variables related to food behaviours (time preparing meals on weekends, purchasing food/beverages at convenience stores, and buying pre-prepared dinners) were considered in Model 3, all previous variables remained significant, and the variable student became negatively associated with TFSs (*p* < 0.05). Spending time preparing meals on weekends (*p* < 0.01) was positively associated with TFSs, while purchasing food/beverages at convenience stores and buying pre-prepared dinners were both negatively associated with TFSs (*p* < 0.001). The highest standardized betas in this model were buying pre-prepared dinners (−0.27), teen meal preparation (0.18), primary cook (0.18), and purchasing food/beverages at convenience stores (−0.18), followed closely by nutrition/cooking course (0.17). Model 3 explained 33.0% of the variance seen in TFSs, an increase of 13% over Model 2.

In Model 4, when variables related to nutrition beliefs were added (preparing healthy food is important, nutritional quality of diet, and nutrition knowledge), the adjusted R^2^ increased to 50%, explaining an additional 17% of the variance over Model 3. All previous variables remained significantly associated with TFSs except gender. Being a student, purchasing food/beverages at convenience stores, and buying pre-prepared dinners were negatively associated with TFSs (*p* < 0.05, *p* < 0.01 and *p* < 0.001, respectively), while the remaining significant variables were positively associated. The relative importance of buying pre-prepared dinners and purchasing food/beverages at convenience stores decreased from Model 3 (standardized betas: −0.27 to −0.14, and −0.18 to −0.10, respectively). Overall, the relative importance of increasing age decreased substantially from Model 1 (0.24) to Model 4 (0.08), and a difference was also seen with primary cook from Model 2 (0.24) to Model 4 (0.11). Nutrition knowledge and nutritional quality of diet were the strongest predictors of TFSs (standardized beta = 0.22, *p*-value < 0.001, for both).

## 4. Discussion

This study assessed food skills in a national sample of adult gym members in Canada. On average, respondents had relatively good food skills, with the majority reporting that they could prepare simple meals from basic ingredients. This is consistent with a study which examined fewer food skills in a larger sample of participants from the Canadian Community Health Survey [37]. In the current study, when it came to more complex skills, such as creating new recipes from scratch, canning food, freezing produce for later use, and dating and labelling stored food, the respondents’ confidence was particularly low. It might be that the frequency of using more advanced skills is lower today than in the past, which in turn, negatively impacts one’s self-efficacy for performing the skill as there are fewer opportunities for social modelling (observing others) and personal mastery (practicing) of the skill [38]. Industrialization and globalization of the food market has also resulted in a wide selection of canned, frozen, and other processed foods that are readily accessible, available year-round and more affordable, and consequently, Canadians are consuming excess processed foods [1]. Similarly, many North Americans are eating away from home daily [4,5,39], with studies in Canada suggesting that on any given day, 20–30% of Canadians eat at a fast-food location, with another 20–30% eating at an ‘other location’ including but not limited to restaurants [5].

To the authors’ knowledge, this is the first study to assess multiple co-variates of TFSs in a Canadian adult population. From the bivariate and multiple regression analyses, it was identified that several factors including demographics, food-related experiences and behaviours, and nutrition-related beliefs were associated with and predicted TFSs. Some variables, including income, education attainment, physical activity, and having children were significantly associated with TFSs in bivariate analyses but were not found to be significant predictors of TFSs when all significant variables were accounted for. In another study from Germany, education level correlated with food skills (r = 0.32) but not cooking skills in a bivariate analysis [40]. It is not known if this relationship would hold if other factors were also considered in the model.

The significant positive association between increasing age and food skills seen in this study might be related to improvements in knowledge and confidence that come with more practice in food preparation [41,42], specifically traditional food preparation, as seen in the UK [42]. In the German study, age was only seen to correlate with cooking skills and not food skills [40], whereas in a Japanese study age, was positively associated with cooking and food skills in females, but inversely associated in males [43]. It should be noted that this study had an age range that included adults up to 80 years, whereas other studies often do not. Overall, one must be cautious with interpretation of age, as our data indicate it was only the largest predictor of TFSs when accounting for demographic variables, whereas the relative contribution of age decreased substantially as more relevant variables were accounted for. Similar results were found with fewer food skills questions in a large group of university students which had a narrower age range [25].

This study also found that females had significantly higher TFSs compared with males, which is consistent with the previous literature from North America, Europe, Australia, and Asia [7,8,18,22,24,25,36,43,44]. Most of this research, however, fails to account for potential covariates, such as nutrition beliefs. Motelli et al. did find that participants with higher cooking and food skills rated the importance of a healthy diet higher, but they did not analyze their results by gender [40]. When nutrition beliefs were included in our blockwise regression, gender was no longer a significant predictor of TFSs. This suggests that a person’s beliefs may have a greater impact than their gender [36,45]. This concept was specifically demonstrated by Lee et al. who found that gender had no effect on the mediating factors of nutrition knowledge and perceived value of healthy food on behavioural intentions [46]. As suggested by many behaviour change theories, perceived knowledge and beliefs act as internal motivators for food-related behaviours [38]. Thus, if the lower food skills that are observed in males are mediated by nutrition-related beliefs, we must find ways to improve the nutrition beliefs in this population. It has been suggested that this work needs to be conducted early, as significant gender differences in nutrition knowledge begin as early as preschool [47]. Qualitative research exploring young males’ perceptions of food skills revealed that most learned these skills from mothers and that greater involvement in food skills occurred with parental encouragement, personal interest, and exposure at school [48]. While the young men acknowledged that gender stereotypes continue to be a barrier, 84% of them supported mandatory education for food skills [49]. It is also noteworthy that male respondents with children had higher TFSs than those with no children. In other studies, motherhood, but not fatherhood, has been associated with improved nutrition behaviours [50,51]. Further studies would be useful to better elucidate this association.

In this study, females who were employed had lower food skills than those who were not; however, this was not observed in males. Previous research with parents (mostly female) observed that planning meals and cooking with whole or basic foods were significantly lower in full-time employed parents; however, after controlling for multiple comparisons, the differences did not remain significant [52].

This study found that being a student was not a significant negative predictor of TFSs until food-related behaviours were accounted for. It is difficult to compare these results to other literature, as many studies examine food skills in student populations, rather than in general adult populations with both students and non-students [23,24,25,26,53].

Primary cooks had significantly higher TFSs than those who were not. This might be related to personal interest, experience, confidence, and knowledge, which are all thought to influence food skills [14,18,48]. In Model 2, being the primary cook was the largest predictor of TFSs after accounting for demographics and food-related experiences, but as more variables were accounted for, the relative contribution decreased.

Those who prepared meals at least four times per week as a teenager had significantly higher TFSs compared with those preparing meals less than once a week as a teenager. Interestingly, there was no significant difference between gender and the frequency of being involved in meal preparation as a teenager. Previous studies found that mothers are most likely to pass on their skills to younger female generations and females are more likely to cook during their teenage years, which is likely due to women traditionally being the primary cook and having more advanced food skills [7,8,10,25,54]. However, both parents are now often pursuing careers outside the home, which results in less time to learn food skills, prepare home-cooked meals, and teach younger generations food skills [54].

The respondents who had taken a nutrition/cooking course had significantly higher TFSs than those who had not. It is probable that taking a nutrition/cooking course would increase one’s knowledge and confidence of food skills [9,23,26]; yet the question remains: how do we motivate those who are not interested to take such courses? Perhaps the solution would be to teach food skills at a young age when they are interested and gender stereotypes are less developed [47,49,55].

Time preparing meals on weekends was positively associated with TFSs. This aligns with another study which found that respondents saved their cooking and meal preparation for the weekend when they had more time [56], an adaptation that likely occurred because more people are working outside the home during the week. Conversely, purchasing food/beverages at convenience stores and buying pre-prepared dinners were negatively associated with TFSs, supporting earlier work with a younger population [25]. Perhaps, those who use time on the weekend to plan and prepare meals for the next week develop higher food skills from practice. Most of the foods available for purchase at convenience stores are pre-packaged and ultra-processed, requiring minimal food skills to prepare, which might explain why we see an association between those who purchase food from these outlets and lower food skills. It is noteworthy that in Model 3, buying pre-prepared dinners was the largest negative predictor of TFSs, but the relative contribution decreased substantially when nutrition-related beliefs were considered.

The addition of nutrition-related beliefs explained an additional 17% of the variance seen in TFSs, with self-reported nutritional quality of diet and nutrition knowledge being the strongest predictors of TFSs. Respondents who rated their nutritional quality of diet and nutrition knowledge higher ate significantly fewer pre-prepared and more home-prepared meals, compared with those with lower scores. These results are supported by another study which demonstrated that nutrition knowledge positively influenced perceived value of healthy foods (β = 0.57); similarly, nutrition knowledge positively influenced behavioural intentions to eat healthy food (β = 0.30) [46]. Thus, it might be that having nutrition knowledge causes one to value the development of food skills as a necessary step in healthier eating. A study by Lavelle et al. on Australian adults found that food skills confidence was a large predictor of diet quality [18]. It appears that if people have nutrition knowledge and value the nutritional quality of their diet they may be motivated to develop food skills, and a cycle will develop whereby improved food skills enable improved dietary quality with less reliance on pre-prepared foods.

The strengths of this study include the recruitment of a geographically diverse population and the use of a previously validated and comprehensive food skills questionnaire to assess food skills [13]. This study also included a variety of potential co-variates and determined 11 factors that influence or predict food skills, providing a foundation for future studies to examine or target these factors in food skills interventions and research.

There are also some limitations. Since this was a cross-sectional study, no direct causations can be concluded from the results and one is not sure which came first, for example, the nutrition beliefs or the food skills. Compared with the national population, this sample had a higher proportion of females, as well as higher income and educational attainment [57]. It is possible that females are more likely to have a personal interest in this topic, and in turn, more female respondents participated. Higher female participation has been observed in other food skills research [13,14,24,25,26,36]. In addition, a greater proportion of this sample exceeds the Canadian 24 h Movement Guidelines [58] compared with the national population [59], confirming they are likely more health conscious. In addition, the overall response rate was low and it is possible that those who self-selected to participate in this study have a greater interest in food skills and perhaps higher food skills scores. Together, these factors could have resulted in a higher mean TFSs score. Nevertheless, the data represent a broad range of respondents and provide a good snapshot of food skills across a wide geographic area and age range. It has also been shown that that there is little evidence of bias from low response rates when using multivariate statistics [60].

## 5. Conclusions

In summary, 11 variables accounted for 50% of the variance seen in TFSs. Increasing age, taking a nutrition/cooking course, participating in meal preparation as a teenager, being a primary cook, time preparing meals on weekends, believing that preparing healthy food is important, and self-reported nutritional quality of diet and nutrition knowledge were positively associated, while being a student, purchasing food/beverages from convenience stores, and buying pre-prepared dinners were negatively associated with food skills. The gender differences observed in TFSs were explained by nutrition-related beliefs (nutrition knowledge and nutritional quality of diet). These nutrition beliefs were the strongest predicators of TFSs overall. These variables should be considered by researchers, dietitians, teachers and others when developing programs to increase food skills in individuals and populations.

## Figures and Tables

**Table 1:** Food Skills Questionnaire (FSQ) item and domain scores based on participants’ ratings (0–100), by primary cook and nutrition/cooking course.

Item ^a^	All Respondents Mean ± SD	Primary Cook Mean ± SD		Nutrition/Cooking Course Mean ± SD	
	(*n* = 576)	Yes (*n* = 468)	No (*n* = 108)	Yes (*n* = 223) ^b^	No (*n* = 348) ^b^
How often do you:					
Check best before dates?	87.3 ± 17.8	**88.2 ± 17.3**	**83.6 ± 19.5 ^¥^**	87.4 ± 16.7	87.6 ± 18.1
Know how the food you eat is grown/produced/processed? ^c^	81.4 ± 20.5	**82.7 ± 20.0**	**75.9 ± 21.9 ^§^**	**85.3 ± 17.0**	**79.0 ± 22.3 ^ǂ^**
Rate your confidence in:					
Budgeting for groceries	75.5 ± 22.8	**77.3 ± 21.8**	**67.7 ± 25.3 ^ǂ^**	**79.2 ± 22.9**	**73.2 ± 22.5 ^§^**
Meal planning	71.0 ± 25.9	**74.0 ± 24.2**	**58.1 ± 29.0 ^ǂ^**	**76.2 ± 23.3**	**67.9 ± 27.0 ^ǂ^**
Selecting vegetables/fruits	86.2 ± 17.9	**88.6 ± 15.2**	**75.5 ± 23.7 ^ǂ^**	**89.9 ± 14.9**	**83.6 ± 19.2 ^ǂ^**
Reading food labels	81.2 ± 22.6	**82.2 ± 21.7**	**76.7 ± 25.9 ^¥^**	**85.5 ± 19.4**	**78.4 ± 24.1 ^ǂ^**
Planning a meal using only foods already in your home	82.4 ± 20.8	**85.0 ± 18.6**	**70.8 ± 25.3 ^ǂ^**	**87.5 ± 16.4**	**79.0 ± 22.6 ^ǂ^**
Adjusting a recipe to make it healthier	76.4 ± 25.0	**79.8 ± 22.5**	**61.5 ± 29.6 ^ǂ^**	**83.3 ± 19.8**	**72.0 ± 27.0 ^ǂ^**
Creating new recipes from scratch ^c^	53.9 ± 33.8	**57.6 ± 33.2**	**37.4 ± 31.6 ^ǂ^**	**60.7 ± 31.4**	**49.2 ± 34.6 ^ǂ^**
What percent of the time do you:					
Use a grocery list?	62.1 ± 33.0	**63.6 ± 32.5**	**55.6 ± 34.5 ^¥^**	62.8 ± 34.2	61.6 ± 32.1
Purchase a variety of vegetables?	78.1 ± 24.6	**79.9 ± 23.1**	**70.1 ± 29.4 ^§^**	80.3 ± 23.1	76.5 ± 25.5
Totals for Food Selection and Planning ^d^	76.0 ± 14.5	**78.1 ± 13.4**	**66.7 ± 15.5 ^ǂ^**	**79.8 ± 12.8**	**73.4 ± 15.0 ^ǂ^**
How many times per week do you make the following home-prepared meals? ^e^					
Breakfast	67.6 ± 37.1	**70.1 ± 36.1**	**56.6 ± 39.6 ^§^**	70.0 ± 36.9	66.1 ± 37.2
Lunch	71.5 ± 26.5	**74.1 ± 25.4**	**60.3 ± 28.6 ^ǂ^**	**74.6 ± 24.7**	**69.4 ± 27.6 ^¥^**
Dinner	72.4 ± 24.4	**74.9 ± 22.2**	**61.2 ± 30.0 ^ǂ^**	**75.1 ± 22.7**	**70.6 ± 25.3 ^¥^**
What percent of the meals you prepare are balanced meals?	75.6 ± 21.5	**78.0 ± 19.8**	**65.1 ± 25.3 ^ǂ^**	**78.3 ± 20.3**	**73.9 ± 22.2 ^¥^**
Overall ability to prepare meals ^ef^	95.1 ± 13.0	**97.1 ± 9.6**	**86.7 ± 20.4 ^ǂ^**	**97.9 ± 8.5**	**93.4 ± 14.9 ^ǂ^**
To what extent do you agree or disagree:					
Food preparation takes too much time ^g^	58.5 ± 29.9	**60.4 ± 30.1**	**49.9 ± 27.2 ^§^**	**63.7 ± 30.4**	**55.1 ± 29.0 ^§^**
Food preparation is too much work ^g^	62.5 ± 29.0	**64.5 ± 29.1**	**53.7 ± 26.7 ^§^**	**66.4 ± 29.3**	**59.9 ± 28.4 ^§^**
Making meals at home helps me to eat healthier	93.3 ± 14.8	94.0 ± 14.0	90.6 ± 17.4	94.5 ± 13.4	92.5 ± 15.6
Food preparation is enjoyable	70.2 ± 26.7	**72.9 ± 25.4**	**58.6 ± 29.3 ^ǂ^**	**73.2 ± 25.0**	**68.2 ± 27.7 ^¥^**
Rate your confidence in:					
Using knives in the kitchen	88.0 ± 16.7	**89.4 ± 15.4**	**81.9 ± 20.2 ^ǂ^**	**90.1 ± 15.3**	**86.6 ± 17.4 ^¥^**
Peeling, chopping and slicing vegetables/fruit	87.8 ± 16.8	**89.9 ± 14.8**	**78.7 ± 21.5 ^ǂ^**	**89.7 ± 16.5**	**86.4 ± 16.9 ^¥^**
Using vegetables in food preparation	88.4 ± 16.9	**91.0 ± 14.0**	**76.9 ± 22.8 ^ǂ^**	**90.8 ± 15.4**	**86.6 ± 17.7 ^§^**
Using beans and lentils in food preparation	62.4 ± 31.1	**65.3 ± 30.2**	**49.7 ± 31.6 ^ǂ^**	**67.6 ± 30.1**	**59.0 ± 31.3 ^§^**
Preparing food from basic ingredients	84.9 ± 19.6	**87.2 ± 18.0**	**75.0 ± 23.0 ^ǂ^**	**89.6 ± 15.8**	**81.7 ± 21.2 ^ǂ^**
Following a basic recipe	88.6 ± 18.6	**90.5 ± 17.7**	**80.6 ± 20.5 ^ǂ^**	**91.7 ± 16.9**	**86.5 ± 19.5 ^§^**
Baking muffins or cake from scratch ^c^	72.6 ± 34.8	**75.7 ± 33.8**	**59.0 ± 35.7 ^ǂ^**	**81.2 ± 28.8**	**67.0 ± 37.2 ^ǂ^**
Boiling, steaming, or stewing	88.2 ± 17.2	**90.2 ± 15.8**	**79.4 ± 20.5 ^ǂ^**	**92.1 ± 14.3**	**85.6 ± 18.5 ^ǂ^**
Stir-frying or pan-frying	86.1 ± 19.9	**87.6 ± 19.3**	**79.9 ± 21.1 ^§^**	**90.3 ± 16.8**	**83.3 ± 21.2 ^ǂ^**
Baking, grilling, or roasting	85.1 ± 20.0	**86.8 ± 19.2**	**77.4 ± 21.9 ^ǂ^**	**89.7 ± 16.8**	**82.0 ± 21.3 ^ǂ^**
Preparing a few dishes at a time to serve all together for a meal ^c^	79.9 ± 23.3	**83.7 ± 20.0**	**63.5 ± 29.1 ^ǂ^**	**86.3 ± 19.4**	**75.7 ± 24.7 ^ǂ^**
Choosing a spice or herb	74.4 ± 24.7	**76.7 ± 23.7**	**64.2 ± 26.7 ^ǂ^**	**79.5 ± 22.0**	**70.9 ± 25.8 ^ǂ^**
Preparing new foods/recipes	74.6 ± 24.6	**78.5 ± 21.6**	**57.8 ± 29.7 ^ǂ^**	**81.1 ± 20.0**	**70.3 ± 26.4 ^ǂ^**
Totals for Food Preparation ^h^	78.5 ± 13.5	**80.8 ± 12.1**	**68.5 ± 14.4 ^ǂ^**	**82.4 ± 12.1**	**75.9 ± 13.7 ^ǂ^**
How often do you:					
Wash countertops before preparing food	78.5 ± 25.0	**80.2 ± 24.3**	**71.4 ± 26.9 ^§^**	80.3 ± 23.8	77.4 ± 25.4
Wash your hands before preparing food	92.5 ± 14.0	**93.1 ± 13.5**	**89.8 ± 15.9 ^¥^**	**94.1 ± 11.7**	**91.4 ± 15.3 ^¥^**
Use microwave/fridge/cold water to thaw frozen meat	78.2 ± 28.1	**80.0 ± 27.2**	**70.3 ± 30.7 ^§^**	**84.6 ± 22.4**	**74.0 ± 30.6 ^ǂ^**
Keep raw meat separate from foods that will not be cooked	90.9 ± 20.5	91.4 ± 20.6	88.9 ± 20.2	**94.2 ± 14.9**	**88.8 ± 23.3 ^§^**
Cook foods to the correct internal temperature	84.0 ± 20.1	**85.9 ± 19.0**	**75.6 ± 22.5 ^ǂ^**	**88.3 ± 14.9**	**81.5 ± 22.1 ^ǂ^**
Wash fruit/vegetables	85.9 ± 21.4	**87.2 ± 20.6**	**80.3 ± 23.9 ^§^**	86.6 ± 20.3	85.4 ± 22.2
Compost fruit/vegetable peelings/waste ^c^	62.6 ± 41.4	62.9 ± 41.6	61.6 ± 40.7	64.8 ± 40.6	61.3 ± 41.8
Put leftovers in the refrigerator within two hours	90.4 ± 15.3	**91.3 ± 14.6**	**86.4 ± 17.4 ^§^**	91.6 ± 13.3	89.5 ± 16.5
Follow the instructions for storage on packaged foods	85.3 ± 20.6	**86.8 ± 19.5**	**78.9 ± 23.9 ^§^**	**87.7 ± 17.7**	**83.8 ± 22.1 ^¥^**
Date and label stored food ^c^	48.7 ± 37.0	48.8 ± 37.5	48.3 ± 35.1	50.4 ± 37.8	47.9 ± 36.4
Check that food is heated throughout when re-heating	81.7 ± 22.0	**83.2 ± 21.2**	**75.3 ± 24.4 ^§^**	**84.1 ± 21.1**	**80.3 ± 22.5 ^¥^**
Rate your confidence in:					
Staying safe in the kitchen (e.g., avoiding burns and cuts)	87.4 ± 15.9	**88.8 ± 14.7**	**81.1 ± 19.2 ^ǂ^**	**89.9 ± 14.8**	**85.7 ± 16.5 ^§^**
Home freezing fruit/vegetables for later use ^c^	64.9 ± 33.5	**66.8 ± 33.4**	**56.6 ± 33.1 ^§^**	**70.8 ± 31.9**	**61.0 ± 34.1 ^§^**
Canning ingredients in sealed jars ^c^	30.8 ± 31.6	31.5 ± 32.0	27.4 ± 29.6	**35.3 ± 31.2**	**27.6 ± 31.3 ^§^**
Totals for Food Safety and Storage ^i^	75.8 ± 12.8	**77.0 ± 12.6**	**70.8 ± 12.1 ^ǂ^**	**78.7 ± 12.3**	**74.0 ± 12.7 ^ǂ^**
Total Food Skills (out of 100)	77.1 ± 11.9	**79.0 ± 11.1**	**68.8 ± 12.0 ^ǂ^**	**80.7 ± 10.9**	**74.7 ± 12.0 ^ǂ^**

^a^ Wording of some questions modified for ease of reporting. ^b^ 5 people did not respond to the question re: previous nutrition/cooking course. ^c^ Eight questions were added to the FSQ [13,22] to enable assessment of more complex food skills. ^d^ Respondent scores were included in domain mean if responses were given to ≥6 of the 11 domain questions. ^e^ Responses were re-weighted out of 100 for consistency with other questions. ^f^ Response categories included: (a) I have no food preparation ability (e.g., heat up pre-prepared foods); (b) I have some food preparation ability (e.g., make sandwiches, salads or scrambled eggs); (c) I can use a combination of pre-prepared ingredients and basic ingredients to prepare homemade meals (e.g., use pre-prepared rotisserie chicken in a home-made casserole); and (d) I can prepare meals from basic ingredients (e.g., make a chicken and vegetable stir-fry with rice). ^g^ Items were reverse coded. ^h^ Respondent scores were included in domain mean if responses were given to ≥11 of the 22 domain questions. ^i^ Respondent scores were included in domain mean if responses were given to ≥7 of the 14 domain questions. Bolded numbers indicate significant differences with ^¥^ *p* < 0.05, ^§^ *p* < 0.01, or ^ǂ^ *p* < 0.001.

**Table 2:** Domain and total food skills by demographic and lifestyle characteristics (*n* = 576).

Characteristics		Total Food Skills		Domain 1: Food Selection and Planning		Domain 2: Food Preparation		Domain 3: Food Safety and Storage	
	Frequency, *n* (%) or Mean ± SD ^¥^	Mean ± SD ^§^	*p*-Value ^ǂ^	Mean ± SD ^§^	*p*-Value ^ǂ^	Mean ± SD ^§^	*p*-Value ^ǂ^	Mean ± SD ^§^	*p*-Value ^ǂ^
**Age (years) (*n* = 557) ^‖^**	41.3 ± 14.8		**<0.001**		**<0.001**		**<0.001**		**<0.001**
18–34	230 (41.3)	73.5 ± 11.4 ^a^		72.1 ± 14.3 ^a^		75.2 ± 13.0 ^a^		71.7 ± 13.1 ^a^	
35–54	204 (36.6)	78.0 ± 11.6 ^b^		76.2 ± 13.9 ^b^		79.6 ± 13.3 ^b^		76.8 ± 11.9 ^b^	
≥55	123 (22.1)	82.5 ± 11.3 ^c^		82.1 ± 13.6 ^c^		83.1 ± 12.9 ^c^		81.8 ± 11.0 ^c^	
**Gender (*n* = 566) ^¶^**			**<0.001**		**<0.001**		**<0.001**		**<0.001**
Female	381 (67.3)	78.9 ± 11.2		77.8 ± 14.1		80.3 ± 12.3		77.4 ± 12.4	
Male	185 (32.7)	73.4 ± 12.6		71.8 ± 14.5		74.9 ± 14.9		72.3 ± 12.9	
**Annual Household Income (CAD) (*n* = 530)**			**0.001**		**0.001 ^∞^**		**0.004**		0.08 *
<60,000	136 (25.7)	73.8 ± 12.2 ^a^		72.0 ± 16.0 ^a^		75.3 ± 13.9 ^a^		72.8 ± 12.5	
60,000–119,999	223 (42.1)	76.9 ± 12.2 ^b^		75.9 ± 14.1 ^ab^		78.4 ± 13.7 ^ab^		75.3 ± 12.8	
≥120,000	171 (32.3)	79.1 ± 11.3 ^b^		78.2 ± 13.2 ^b^		80.6 ± 12.8 ^b^		77.5 ± 13.0	
**Education (*n* = 572)**			**<0.001**		**<0.001 ^∞^**		**<0.001 ^∞^**		**0.03**
Some high school or high school	72 (12.6)	72.5 ± 12.4 ^a^		70.9 ± 15.7 ^a^		73.6 ± 14.4 ^a^		72.2 ± 13.1 ^a^	
College, trade or undergraduate	354 (61.9)	77.0 ± 12.2 ^b^		75.4 ± 14.9 ^a^		78.3 ± 13.6 ^b^		76.0 ± 12.8 ^ab^	
Graduate or professional	146 (25.5)	79.8 ± 10.5 ^c^		79.8 ± 11.8 ^b^		81.6 ± 11.9 ^c^		77.0 ± 12.3 ^b^	
**Children (*n* = 569)**			**0.004**		0.11		0.08 *		**<0.001**
No	420 (73.8)	76.3 ± 12.4		75.4 ± 15.2		77.8 ± 13.9		74.7 ± 13.1	
Yes	149 (26.2)	79.4 ± 10.4		77.4 ± 12.4		80.6 ± 12.1		79.0 ± 11.2	
**Living Situation (*n* = 569)**			**<0.001**		**<0.001**		**<0.001**		0.05 *
Dependently, with parents/family	83 (14.6)	70.6 ± 13.7		67.9 ± 16.4		70.7 ± 16.0		72.4 ± 14.0	
Independently, from parents/family	486 (85.4)	78.3 ± 11.3		77.3 ± 13.7		79.9 ± 12.5		76.4 ± 12.4	
**Student (*n* = 568)**			**0.005**		0.07		0.14 *		0.10 *
No	493 (86.8)	77.6 ± 11.7		76.3 ± 14.2		79.0 ± 13.2		76.4 ± 12.3	
Yes	75 (13.2)	73.5 ± 13.3		73.1 ± 16.1		75.2 ± 15.1		71.1 ± 14.7	
**Currently Employed (*n* = 572)**			**<0.001**		**<0.001**		**0.01**		**<0.001**
No	110 (19.2)	80.8 ± 12.9		80.8 ± 14.8		81.4 ± 14.3		79.8 ± 13.7	
Yes	462 (80.8)	76.3 ± 11.6		74.8 ± 14.2		77.9 ± 13.2		74.9 ± 12.4	
**Living with a Health Condition (*n* = 572)**			**0.04**		**0.004**		**0.03 ^†^**		0.26
No	440 (76.9)	76.6 ± 12.1		75.0 ± 14.8		78.0 ± 13.5		75.5 ± 12.8	
Yes	132 (23.1)	79.0 ± 11.2		79.1 ± 13.0		80.3 ± 13.4		76.9 ± 12.4	
**Primary Cook (*n* = 575)**			**<0.001**		**<0.001**		**<0.001**		**<0.001**
No	108 (18.6)	68.8 ± 12.0		66.7 ± 15.5		68.5 ± 14.4		70.8 ± 12.1	
Yes	468 (81.4)	79.0 ± 11.1		78.1 ± 13.4		80.8 ± 12.1		77.0 ± 12.6	
**Nutrition/Cooking Course (*n* = 571)**			**<0.001**		**<0.001**		**<0.001**		**<0.001**
No	348 (60.9)	74.7 ± 12.0		73.4 ± 15.0		75.9 ± 13.7		74.0 ± 12.7	
Yes	223 (39.1)	80.7 ± 10.9		79.8 ± 12.8		82.4 ± 12.1		78.7 ± 12.3	
**Teen Meal Preparation (times/wk) (*n* = 569)**			**<0.001**		**0.002**		**<0.001 ^∞^**		0.11 *
<1	250 (43.9)	75.2 ± 12.6 ^a^		74.1 ± 14.8 ^a^		76.1 ± 14.7 ^a^		74.6 ± 13.2	
1–3	211 (37.1)	77.5 ± 11.1 ^ab^		75.8 ± 14.1 ^a^		79.3 ± 11.7 ^b^		75.8 ± 12.6	
≥4	108 (19.0)	80.6 ± 11.1 ^b^		79.9 ± 13.5 ^b^		82.3 ± 12.8 ^b^		78.5 ± 11.3	
**Purchasing Food/Beverages at Convenience Stores (*n* = 572)**			**<0.001**		**<0.001**		**<0.001**		**<0.001**
No	293 (51.2)	80.7 ± 11.1		80.3 ± 13.1		82.2 ± 12.5		78.8 ± 12.2	
Yes	279 (48.8)	73.3 ± 11.7		71.3 ± 14.5		74.6 ± 13.4		72.7 ± 12.5	
**Purchasing Food/Beverages at Grocery stores/Farmers’ Markets/Bakeries (CAD/mon) (*n* = 571)**			**0.002**		0.14 *		0.09 *		**<0.001**
1–299	198 (34.7)	75.7 ± 11.7 ^a^		74.1 ± 15.0		77.5 ± 13.1		74.2 ± 12.2 ^a^	
300–499	191 (33.5)	76.1 ± 11.9 ^a^		75.8 ± 14.2		77.4 ± 13.7		74.3 ± 12.7 ^a^	
≥500	182 (31.9)	79.6 ± 11.9 ^b^		78.1 ± 13.9		80.7 ± 13.6		79.1 ± 12.8 ^b^	
**Purchasing Food/Beverages at Restaurants/Fast Food/Coffee Shops (CAD/mon) (*n* = 560)**			**<0.001**		**<0.001**		**<0.001**		**0.005**
1–99	211 (37.7)	80.2 ± 11.5 ^a^		79.3 ± 14.1 ^a^		82.0 ± 12.8 ^a^		77.9 ± 12.5 ^a^	
100–199	173 (30.9)	76.0 ± 12.2 ^b^		74.9 ± 14.7 ^b^		77.6 ± 13.2 ^b^		74.4 ± 13.3 ^b^	
≥200	176 (31.4)	74.0 ± 11.3 ^b^		72.4 ± 14.1 ^b^		74.6 ± 13.4 ^b^		74.3 ± 12.0 ^b^	
**Time Preparing Meals (weekday min/d) (*n* = 562)**			**<0.001**		**<0.001**		**<0.001 ^∞^**		**0.001**
1–29	164 (29.2)	72.8 ± 12.7 ^a^		71.2 ± 15.4 ^a^		73.6 ± 15.3 ^a^		72.7 ± 12.2 ^a^	
30–59	286 (50.9)	78.9 ± 11.3 ^b^		77.7 ± 13.7 ^b^		80.6 ± 12.0 ^b^		77.1 ± 13.0 ^b^	
≥60	112 (19.9)	79.3 ± 10.9 ^b^		78.8 ± 13.3 ^b^		81.1 ± 12.1 ^b^		76.9 ± 12.2 ^b^	
**Time Preparing Meals (weekend min/d) (*n* = 557)**			**<0.001 ^∞^**		**<0.001**		**<0.001 ^∞^**		0.19
1–29	86 (15.4)	73.0 ± 13.4 ^a^		72.7 ± 15.7 ^a^		72.7 ± 16.2 ^a^		73.6 ± 12.8	
30–59	226 (40.6)	76.7 ± 11.2 ^ab^		75.0 ± 14.0 ^a^		77.9 ± 12.4 ^b^		76.1 ± 12.4	
≥60	245 (44.0)	79.6 ± 11.0 ^c^		78.9 ± 12.9 ^b^		81.9 ± 11.6 ^c^		76.5 ± 13.0	
**Grocery Shopping (times/wk) (*n* = 574)**			**<0.001**		**<0.001**		**<0.001**		0.12
<1	52 (9.1)	72.8 ± 11.5 ^a^		69.7 ± 15.3 ^a^		74.1 ± 13.0 ^a^		73.0 ± 12.7	
1	271 (47.2)	76.1 ± 12.3 ^a^		74.6 ± 15.1 ^a^		77.1 ± 14.0 ^a^		75.5 ± 12.6	
≥2	251 (43.7)	79.2 ± 11.3 ^b^		78.7 ± 13.0 ^b^		80.9 ± 12.5 ^b^		76.8 ± 12.9	
**Buying Pre-prepared Breakfast (times/wk) (*n* = 571)**			**<0.001**		**<0.001**		**<0.001**		**0.007**
0	396 (69.4)	78.7 ± 12.1 ^a^		77.8 ± 14.5 ^a^		80.3 ± 13.6 ^a^		76.8 ± 12.9 ^a^	
1	94 (16.5)	76.1 ± 10.1 ^a^		74.2 ± 12.0 ^a^		77.4 ± 11.1 ^a^		75.3 ± 11.4 ^ab^	
≥2	81 (14.2)	70.9 ± 11.3 ^b^		69.1 ± 14.9 ^b^		71.2 ± 12.7 ^b^		72.0 ± 12.7 ^b^	
**Buying Pre-prepared Lunch (times/wk) (*n* = 571)**			**<0.001**		**<0.001**		**<0.001**		**<0.001**
0	246 (43.1)	80.5 ± 10.9 ^a^		79.7 ± 13.3 ^a^		82.5 ± 11.9 ^a^		77.9 ± 13.0 ^a^	
1	146 (25.6)	76.5 ± 11.4 ^a^		77.6 ± 13.6 ^a^		79.8 ± 13.0 ^a^		77.1 ± 11.4 ^a^	
≥2	179 (31.3)	71.5 ± 11.8 ^b^		69.5 ± 14.7 ^b^		72.0 ± 13.6 ^b^		72.1 ± 12.5 ^b^	
**Buying Pre-prepared Dinner (times/wk) (*n* = 570)**			**<0.001**		**<0.001 ^∞^**		**<0.001 ^∞^**		**<0.001**
0	111 (19.5)	83.9 ± 10.4 ^a^		83.4 ± 13.1 ^a^		86.7 ± 10.7 ^a^		79.8 ± 12.8 ^a^	
1	230 (40.4)	78.3 ± 10.7 ^b^		77.5 ± 13.0 ^b^		80.0 ± 11.6 ^b^		76.3 ± 12.2 ^b^	
≥2	229 (40.2)	72.7 ± 12.1 ^c^		70.9 ± 14.7 ^c^		73.0 ± 14.1 ^c^		73.7 ± 12.8 ^b^	
**Moderate to Vigorous Physical Activity (min/wk) (*n* = 572)**			**0.003**		**0.001**		**<0.001**		0.88
<90	97 (17.0)	73.8 ± 11.2 ^a^		71.4 ± 15.0 ^a^		74.0 ± 12.4 ^a^		75.2 ± 11.9	
90–149	155 (27.1)	76.7 ± 11.1 ^ab^		75.3 ± 14.4 ^ab^		77.8 ± 12.9 ^ab^		76.0 ± 12.4	
≥150	320 (55.9)	78.4 ± 12.4 ^b^		77.6 ± 14.1 ^b^		80.2 ± 13.8 ^b^		75.9 ± 13.2	

^¥^ Sample size for statistical analyses with total food skills; sample size varied by question as ethics required that respondents were able to skip questions. ^§^ Column means with different superscript letters are significantly different in post hoc analyses. ^ǂ^ Categorical independent variables were analyzed using independent samples *t*-test when two groups were compared and one-way ANOVA when 3 groups were compared. Bolded *p*-values indicate significant differences. ^‖^ Age was also categorized as young, middle-aged, and older adults to assess the level of food skills in these particular groups as this may be useful for program planning. **^¶^** Two respondents who identified as other were excluded as sample size was too small for comparisons with other groups. * K-sample Median test or Mann–Whitney test was used because data were skewed and/or non-normally distributed; none of these results were significant. Means are presented for consistency. ^†^ Mann–Whitney test was used because normality assumption was violated. **^∞^** Welch’s statistic and Games Howell Post Hoc was used to correct for unequal variances.

**Table 3:** Correlation of nutrition-related beliefs by domain and total food skills (*n* = 576).

Variable	Pearson Correlation Coefficient			
	Total Food Skills	Domain 1: Food Selection and Planning	Domain 2: Food Preparation	Domain 3: Food Safety and Storage
**Importance of Preparing Healthy Food (*n* = 529)**	0.51 ^ǂ^	0.52 ^ǂ^	0.47 ^ǂ^	0.37 ^ǂ^
**Nutritional Quality of Diet (*n* = 573)**	0.56 ^ǂ^	0.52 ^ǂ^	0.52 ^ǂ^	0.41 *^ǂ^
**Nutrition Knowledge (*n* = 572)**	0.58 ^ǂ^	0.63 ^ǂ^	0.53 ^ǂ^	0.41 *^ǂ^

^ǂ^ Pearson’s correlation *p* < 0.001. * Spearman’s correlation was used because data were not normally distributed.

**Table 4:** Predictors of food skills in gym members (*n* = 470).

Characteristics	Model 1 ^a^		Model 2 ^b^		Model 3 ^c^		Model 4 ^d^	
	*B* ^e^	*β* ^f^	*B* ^e^	*β* ^f^	*B* ^e^	*β* ^f^	*B* ^e^	*β* ^f^
Age	3.72 ^ǂ^	0.24	3.29 ^ǂ^	0.22	1.66 ^¥^	0.11	1.17 ^¥^	0.08
Male	−4.63 ^ǂ^	−0.19	−2.73 ^¥^	−0.11	−2.15 ^¥^	−0.09	−0.73	−0.03
Student	−0.81	−0.02	−1.21	−0.04	−2.80 ^¥^	−0.08	−2.82 ^¥^	−0.08
Primary Cook			7.24 ^ǂ^	0.24	5.48 ^ǂ^	0.18	3.42 ^§^	0.11
Teen Meal Preparation			2.60 ^ǂ^	0.17	2.84 ^ǂ^	0.18	2.15 ^ǂ^	0.14
Nutrition/Cooking Course			3.84 ^ǂ^	0.16	4.05 ^ǂ^	0.17	2.63 ^§^	0.11
Time Preparing Meals (Weekend)					1.68 ^§^	0.10	1.42 ^¥^	0.09
Purchasing Food/Beverages at Convenience Stores					−4.26 ^ǂ^	−0.18	−2.31 ^§^	−0.10
Buying Pre-prepared Dinners					−4.22 ^ǂ^	−0.27	−2.22 ^ǂ^	−0.14
Preparing Healthy Food is Important							0.11 ^§^	0.13
Nutritional Quality of Diet							0.15 ^ǂ^	0.22
Nutrition Knowledge							0.16 ^ǂ^	0.22
Constant ^g^	75.83		66.17		72.00		37.55	
Adjusted R^2 h^	0.09		0.20		0.33		0.50	

^a^ Model 1, demographic characteristics (age, gender, student). ^b^ Model 2, Model 1 plus variables related to food experiences (primary cook, teen meal preparation, nutrition/cooking course). ^c^ Model 3, Model 2 plus variables related to food behaviours (time preparing meals on weekends, purchasing food/beverages at convenience stores, buying pre-prepared dinner). ^d^ Model 4, Model 3 plus variables related to nutrition beliefs (preparing healthy food is important, nutritional quality of diet, nutrition knowledge). ^e^ *B* is the unstandardized beta coefficient that represents the change in the total food skills score for one unit of change in the predicator variable. ^f^ *β* is the standardized beta coefficient that represents the influence of the variable on total food skills score when taking into consideration all variables in the model. ^g^ Constant is the y-intercept, which is the excepted mean value in total food skills score when all the predictor variables equal zero. ^h^ Adjusted R^2^ is the proportion of variance in the total food skills score that is explained by the predictor variables in the model when taking into account the other variables included in the model. ^¥^ *p* < 0.05, ^§^ *p* < 0.01, ^ǂ^ *p* < 0.001.

## Data Availability

The data presented in this study are available on request from the corresponding author.
